# The need for trained graduates to promote exercise as a mode of preventative healthcare in the United Arab Emirates

**DOI:** 10.3389/fspor.2025.1682177

**Published:** 2025-11-19

**Authors:** Paul Stapley, Jean-Pierre Bailey, Khalil Yousef, Romuald Lepers, Suzanne Robertson-Malt

**Affiliations:** 1 School of Medical, Indigenous and Health Sciences, University of Wollongong, Wollongong, NSW, Australia; 2 Institute for Healthier Living Abu Dhabi, Al Nahyan, Abu Dhabi, United Arab Emirates; 3 School of Humanities, Social Sciences and Health, University of Wollongong Dubai, Dubai, United Arab Emirates; 4 School of Nursing, The University of Jordan, Amman, Jordan; 5 Université Bourgogne Europe, INSERM, CAPS UMR 1093, Dijon, France

**Keywords:** physical activity, cardiovascular, obesity, cancer, sedentary, exercise physiology

## 1 Introduction

The United Arab Emirates (UAE) has seen a rapid increase in population through natural growth and net migration from 3.2 million people in 2000 to 10.7 million in 2023 (1). Current life expectancy is 79.9 years for males and 81.4 years for females, but average healthy life expectancy (“healthspan”) is 66 years (2). While it has made very significant advances in healthcare modernization and infrastructure the UAE is experiencing a growing health crisis linked to lifestyle-related diseases, physical inactivity and obesity which is compounded by a lack of integration of trained exercise professionals into clinical, community, and educational workforce settings. Here, we advocate that the UAE must embark on developing structured pathways for graduates in exercise physiology, kinesiology, and rehabilitation sciences and rapidly upscale its capacity to train and deploy qualified professionals to deliver preventive, exercise and movement-based care across the lifespan.

## 2 Physical inactivity and obesity in the UAE

Eight-five per cent of the UAE population reside in urban areas and lead sedentary lifestyles. Nash et al. (3) reported that only 42.1% of males and 28.4 of females meet internationally recommended physical activity (PA) levels compared to global averages of 76.6% and 68.3% (males and females, respectively). Abusnana et al. (4) documented that in 2010 25% of Emirati men and 40% of women were obese [a Body Mass Index, BMI of >30 kg/m^2^ (5)]. Between 2000 and 2018, obesity rates in the UAE doubled from 16% to 34% in males and females and severe obesity rose from 2% to 11%. From a sample of 6,789 patient records, Radwan et al. (6) also documented a two to three-fold increase in the prevalence of obesity between 1989 and 2017.

Children in the UAE show particularly concerning levels of overweight or obesity with only 19% of school-aged children meeting the recommended 60 min of daily moderate to vigorous levels of PA (7). Youth in the UAE spend an estimated 80% of their waking hours in sedentary behaviours, and active transport (e.g., walking or cycling to school) remains rare (8). Al-Haddad et al. (9) documented that from 16,391 children in the UAE, BMI measures indicated a greater risk of being overweight than international norms. At 10 years of age, UAE children were 1.7 times more likely to be overweight and 1.9 times more likely at 19 years of age (10). A study by Albooshi et al. (11) of 44,942 students’ BMI's suggested that rates of obesity had risen to 41.2% in boys and girls between 11 and 14 years of age and obesity increases linearly by 2.4% in children in the UAE between 3 and 12 years of age. Such high rates of obesity and inactivity in UAE children and youth leaves them susceptible to chronic diseases, in later life.

## 3 Cardiovascular disease and cancer in the UAE

Cardiovascular disease (CVD) is the leading cause of mortality in the UAE (12). Short term exercise has important effects on reducing CVD risk, improving body composition and overall cardiorespiratory fitness. One preventable cause of CVD is hypertension. For every 20 mmHg and 10 mmHg increase in systolic and diastolic blood pressure respectively, the chances of heart disease and stroke are doubled. Bhagavthula et al. (13) reported hypertension in 31% of the UAE population (37% in Dubai) from a total of 139,907 participants measured. Changing the prevalence of CVD and related conditions such as hypertension requires a change in lifestyle, greater levels of PA along with an improved diet. Indeed, Dalibata et al. (14) showed that an 8-week exercise intervention consisting of 60 min, three times per week, moderate to high intensity exercise was sufficient to improve biochemical parameters and overall fitness measures in a group of UAE students.

Cancer has been identified as the second leading cause of mortality in the UAE (15) with an incidence in 2012 of 92.5/100,000 people (16). A systematic review of studies conducted between 2007 and 2016 (17) cited a lack of physical activity (alongside tobacco use and an unhealthy diet) as a significant risk factor for cancer in the UAE population. Even if it is unclear whether exercise adherence is simply a marker of a generally more active lifestyle, leads to biological effects that counter cancer initiation or progression, or if it increases the effectiveness or tolerance to cancer treatments, there is ample evidence that higher levels of regular exercise are associated with a lower risk of developing cancer and improving the prognosis and mortality rates in cancer patients (18).

## 4 The workforce needs to promote health and longevity in the UAE through exercise

Physically inactive individuals lose an estimated 3–5 quality-adjusted life years and experience up to 7–10 fewer years of disease-free living compared to their active counterparts (19). Moreover, given the public health issues outlined above, the fact that the UAE currently has no clear standards or credentialing pathways to train graduates that can deliver evidence-based exercise interventions is a matter requiring urgent attention and is largely dependent on ex-patriate fitness professionals who currently promote regular PA and exercise. When coupled with the treatment of the acute and chronic effects of disease, the strategy of increasing PA adherence could help to reduce the occurrence of lifestyle-related diseases and lessen the overall burden on the healthcare system of the UAE. Addressing these issues requires effective, evidence-based interventions to promote a healthy lifestyle for the UAE population. Such interventions can best be implemented by trained exercise scientists who possess the specialized skills to design evidence-based physical activity programs tailored to individual needs, effectively and safely preventing and managing chronic conditions. Graduates in exercise science are adept at assessing the physical limitations of individuals or patients and delivering interventions that respect those limits, avoiding compromises to cardiorespiratory or musculoskeletal health. This unique expertise enables them to guide individuals toward sustainable health improvements safely and effectively (20).

Alongside significant investments in infrastructure and initiatives to encourage participation in sports and exercise, the UAE will require skilled graduates trained in all aspects of PA and exercise science with basic and applied knowledge of human physiology, exercise assessment and prescription, dietary monitoring, data logging and retrieval, and human nutrition. Educational programs, notably in Australia, New Zealand, Canada, UK and the USA offer accredited under- and post-graduate degrees in exercise and sports science (21). International standards to include a larger range of so far under-represented countries such as Greece, Portugal, and Spain are currently the subject of a collaborative network (21). Graduates are trained to work in the community and the regulated private sector for the promotion of health through PA and sports as a preventive strategy against disease, one recently recognised by the ‘Global Alliance for the Promotion of Physical Activity’ by 139 countries (22). In the face of a growing, large sedentary population the UAE must embark on a transformative strategy of exercise adherence and aligning with this alliance.

Critically, professionals trained in clinical exercise physiology, behavioural health change, and long-term rehabilitation planning are also scarce in the UAE. While policy documents acknowledge the emerging role of exercise professionals in public health and chronic disease prevention, their practical implementation remains limited. The lack of graduate-level programs and credentialing pathways restricts the country's ability to scale evidence-based interventions. Unlike countries with mature exercise medicine ecosystems, the UAE does not yet offer national licensure for clinical exercise physiologists. This absence limits the ability of hospitals, schools, and primary care settings to integrate these professionals into routine care. Practical implementation may require overcoming several potential challenges including the establishment of national standards, securing University partners and developing under- and postgraduate programs, and defining clear career pathways for graduates and focused messaging for the UAE context. Implementation pathways for exercise science standards and professional licensing could be established through a national advisory board composed of academics, exercise science professionals and industry representatives.

## 5 Conclusion: building capacity for a healthier future in the UAE

The UAE must invest in the development and deployment of a qualified exercise health workforce. Graduate programs in clinical exercise physiology, rehabilitation sciences, and preventive health must be embedded in academic institutions, supported by licensure frameworks and integrated into healthcare delivery. Exercise is no longer optional but is a central pillar of modern medicine. Without trained professionals to implement movement-based care, the promise of preventive healthcare and longevity cannot be realized. By cultivating this next generation of health professionals, the UAE can lead the Middle East in advancing population health through structured, evidence-based physical activity interventions.
